# Multi-platform metagenomic characterization of the microbial community during spontaneous cacao fermentation

**DOI:** 10.3389/fbioe.2025.1630515

**Published:** 2025-08-26

**Authors:** Joel Tigrero-Vaca, Mirian Villavicencio-Vásquez, Jonathan Coronel, Juan Manuel Cevallos-Cevallos

**Affiliations:** ^1^ Escuela Superior Politécnica del Litoral, ESPOL, Centro de Investigaciones Biotecnológicas del Ecuador (CIBE), Guayaquil, Ecuador; ^2^ Escuela Superior Politécnica del Litoral (ESPOL), Facultad de Ingeniería Mecánica y Ciencias de la Producción (FIMCP), Centro de Biotecnología (CIBE), Guayaquil, Ecuador

**Keywords:** fine flavor cacao, Illumina, Nanopore, food biotechnology, metagenomics

## Abstract

Cacao fermentation is a spontaneous process in which microorganisms play a key role in the development of distinctive chocolate flavors. The microbiota acting during cacao fermentation has been routinely characterized by culture-based techniques and next-generation sequencing using Illumina’s platform. However, the potential of *in situ* sequencing technologies to monitor microbial dynamics during cacao fermentation has not been assessed. In this study, cacao bean samples were collected at 0, 24, 48, 72, and 96 h after the start of the fermentation. Total DNA was extracted, and sequencing libraries were prepared for further sequencing using Illumina’s and Nanopore’s MinION sequencing platforms. Additionally, microorganisms were isolated using traditional culture-based methods. At the order and family taxonomic levels, Illumina and MinION sequencing revealed similar microbial composition in the samples. However, discrepancies were observed at the genus and species levels. In this sense, Illumina sequencing revealed a predominance of *Limosilactobacillus*, *Levilactobacillus*, *Lactiplantibacillus, Frauteria*, *Saccharomyces* and *Acetobacter*, while MinION sequencing showed a prevalence of *Escherichia*, *Salmonella*, *Liquorilactobacillus*, *Lentilactobacillus*, *Acetobacter* and *Komagataeibacter* during fermentation. The three methods were consistent in detecting the major yeast (*Saccharomyces cerevisiae*), lactic acid bacteria (*Lactiplantibacillus plantarum*, *Leuconostoc pseudomesenteroides*, *Levilactobacillus brevis*, *Liquorilactobacillus mali*, and *Lentilactobacillus hilgardii*) and acetic acid bacteria (*Acetobacter pasteurianus*) species during fermentation. Functional analysis based on a hybrid assembly of Illumina and MinION data revealed the roles of lactic acid bacteria and acetic acid bacteria in the metabolism of carbohydrates, amino acids, and secondary metabolites such as polyphenols and theobromine. This study represents the first report assessing the applicability of MinION sequencing for the characterization of microbial populations during cacao fermentation, demonstrating its potential as a complementary tool to established sequencing platforms.

## 1 Introduction

Cacao beans are the main raw material used for chocolate production (Schwan et al., 2014), and fermentation of the raw beans is a critical step in the formation of flavor compounds through a cascade of enzymatic and biochemical processes (Santander Muñoz et al., 2020).

Most of the world’s cacao production is linked to the Forastero variety, which is regarded as bulk cacao because of its strong basic cacao character (Rottiers et al., 2019a). Criollo and Trinitario are considered as fine flavor and are widely utilized for the elaboration of specialty chocolate; these varieties are mostly cultivated in South America and Asia (Laliberté et al., 2012; Kongor et al., 2016). Nacional cacao genotype is mostly produced in Ecuador and is characterized by its strong floral notes. Nonetheless, materials of pure Nacional cacao are scarce since the hybrids between Nacional and Trinitario have become prevalent in Ecuador (Samaniego et al., 2020). However, the Nacional x Trinitario complex maintains the unique traits of fine cacao (Rottiers et al., 2019b; Tigrero-Vaca et al., 2022). Beyond their sensory qualities, these fine flavor varieties also command higher economic value in the global cacao market (Parra, 2017).

In addition to genetic factors, post-harvest processing, particularly fermentation, plays a crucial role in determining cocoa bean quality by facilitating the development of flavor precursors (Streule et al., 2024). Years of research led to the conclusion that a proper cacao fermentative process requires a succession of specific yeasts, lactic acid bacteria (LAB) and acetic acid bacteria (AAB) (De Vuyst and Weckx, 2016). Within this frame, most of the studies have utilized amplicon analysis for profiling microbial communities present during Forastero, Criollo and Trinitario cacao fermentation (Camu et al., 2007; Garcia-Armisen et al., 2010; Lefeber et al., 2011; Díaz-Muñoz et al., 2021). In recent years, only a few studies have employed shotgun metagenomic sequencing for assessing the microbial communities during fermentation of Trinitario (Verce et al., 2021), Forastero and Criollo cultivars (Carolina et al., 2021; Mota-Gutierrez et al., 2021). To date, however, no such studies have been conducted on the fermentation of Nacional × Trinitario beans.

Microbial activity during fermentation has traditionally been studied through culture-dependent techniques (Camu et al., 2007; Lagunes Gálvez et al., 2007; Nielsen et al., 2007) and more recently, through next-generation sequencing (NGS) techniques (Mota-Gutierrez et al., 2018; 2021; Verce et al., 2021). Among these, amplicon sequencing, targeting the 16S rRNA gene for bacteria and the internal transcribed spacer (ITS) region for fungi, has been widely used to analyze microbial communities (Nilsson et al., 2019; Gao et al., 2021). However, this approach can introduce bias due to differential amplification efficiencies, which may distort relative abundance estimates (Bista et al., 2018).

Shotgun metagenomic sequencing offers a robust alternative by analyzing the total genomic content of all microorganisms present in a sample without relying on targeted genetic markers (Ranjan et al., 2016). This method provides a more comprehensive and accurate taxonomic resolution, capturing even rare and low-abundance species down to the species level (Brumfield et al., 2020).

Traditionally, shotgun metagenomics has been performed using Illumina platforms due to their high sequencing accuracy and reliability (Serra et al., 2019). However, these platforms are limited by short read lengths, which can introduce analytical bias in complex microbial communities (Kim et al., 2011), and they require extensive laboratory infrastructure, restricting their use in field-based studies.

Oxford Nanopore Technologies (ONT)’s portable MinION sequencer offers a promising alternative. It generates long reads that enable more detailed and contiguous microbial characterization (Shin et al., 2018) and has been applied to diverse studies ranging from bacterial genotyping to antibiotic resistance profiling (Brown et al., 2017; Quick et al., 2017; Kai et al., 2019). Nonetheless, nanopore sequencing has not yet been utilized to investigate microbial communities during the fermentation of Nacional × Trinitario cacao beans.

Understanding the microbial dynamics involved in cacao fermentation is vital for improving post-harvest practices and enhancing bean quality. In this context, the objective of this study was to characterize the microbial communities involved in the fermentation of Nacional × Trinitario cacao beans using both culture-dependent methods and NGS technologies, including Illumina and Oxford Nanopore sequencing platforms.

## 2 Materials and methods

### 2.1 Sample collection

Samples of Nacional x Trinitario cacao beans were collected from a farm located in the Guayas province of Ecuador. Fermentation was carried out in the farm using 0.06 m^3^ wooden boxes containing roughly 1,000 kg of cacao beans. About 100 g of cacao beans were collected at 0 h, 24 h, 48 h, 72 h, and 96 h after the beginning of the spontaneous fermentation process and stored at −80 °C until analyzed. Three fermentation replicates were run for this study.

### 2.2 Culture based microbiological assay

A traditional microbiological analysis was performed on each sample. For this purpose, 5 g of sample were homogenized in 10 mL of peptone water (TM Media, Bhiwadi, India). Subsequently, 1 mL of the homogenate was used to prepare six serial decimal dilutions. After this, 100-μL aliquots of the dilutions were spread in each of the following culture media: Potato Dextrose Agar (PDA, TM Media, Bhiwadi, India) supplemented with 1 μg/mL of tetracycline and incubated for 5 days at 30 °C for yeast isolation; Man-Rogosa-Sharpe (MRS, TM Media, Bhiwadi, India) agar incubated at 30 °C for 2 days for the growth of lactic acid bacteria; and Glucose Yeast Calcium Carbonate (GYC) medium (Sm et al., 2010), composed of 10% glucose, 1% yeast extract, 2% calcium carbonate and 1.5% agar (pH 6.8), incubated at 30 °C for 4 days for the isolation of acetic acid bacteria.

After incubation, 15 representative colonies were selected based on distinct morphological traits, including colony color, size, shape, margin, texture, and opacity, to capture a range of culturable microbial diversity (Sousa et al., 2013). This approach ensured a phenotypically diverse subset of colonies for downstream isolation, even within labor constraints. The selected colonies were subcultured onto fresh plates of the respective media (PDA, MRS, or GYC). The isolates were incubated for 4–5 days to obtain pure cultures. Colony morphology and Gram staining were used for preliminary identification, following the protocols described by Public Health England (2019).

### 2.3 DNA extraction from microbial isolates

DNA extraction from isolates was performed as proposed in a previous study (Dashti and Dashti, 2009) with some modifications. Briefly, single colonies were picked using a loop and dissolved by vortexing in 20 μL of 20 mM NaOH. After this, the suspensions were heated on a microwave at high intensity (1,000 W) for 90 s. The quantity and quality of the DNA extracted from the microbial isolates was verified by spectrophotometry (NanoDrop; Thermo Fisher Scientific, Wilmington, DE, United States). The extracted DNA was submitted to PCR amplification as indicated below.

### 2.4 Culture dependent assessment of microbial communities

For bacterial isolates, the primers 27F (5′AGA​GTT​TGA​TCC​TGG​CTC​AG3′) and 1492R (5′GGT​TAC​CTT​GTT​ACG​ACT​T3′) were used for the amplification of the 16S rRNA region of bacterial DNA, as described by Zhang et al. (2012). The PCR reaction was prepared in a final volume of 15 μL, containing 7.5 μL of GoTaq Green Master Mix (Promega Corporation, Madison, WI, United States), 1 μL of each primer, 5.5 μL of ultrapure sterile distilled water, and 1 μL of bacterial DNA. PCR amplification was carried out in a Mastercycler thermocycler (Eppendorf Nexus GSX1, Hamburg, Germany) using the following conditions: initial denaturation at 95 °C for 15 min; 30 cycles of denaturation at 95 °C for 1 min, annealing at 54 °C for 1 min, and extension at 72 °C for 2 min; followed by afinal extension of 72 °C for 10 min, as proposed by Galkiewicz and Kellogg (2008).

For yeasts isolates, the internal transcribed spacer (ITS) region was amplified using the ITS1 (5′CTG​GGT​CAT​TTA​GAG​GAA​GTA​A3′) and ITS4 (5′TCC​TCC​GCT​TAT​TGA​TAT​GC3′) primers as described by Fujita et al., 2001. The PCR reaction was prepared in a final volume of 15 μL containing 7.5 μL of GoTaq Green Master Mix (Promega Corporation, Madison, WI, United States), 1 μL of each primer, 5.5 μL of sterile ultrapure distilled water. and 1 μL of yeast DNA. The amplification consisted of an initial denaturation at 94 °C for 7 min, 30 cycles of denaturation at 94 °C for 45 s, annealing at 55 °C for 1 min, and extension at 72 °C for 1 min; followed by a final extension at 72 °C for 7 min.

The presence of bacterial and yeast amplicons was confirmed by agarose gel electrophoresis. PCR products were subsequently sent to an external laboratory for Sanger sequencing. The taxonomic identification of the microbial isolates was carried out by aligning the sequence data to the GenBank database using BLAST (Basic Local Alignment Search Tool) in Geneious Prime program version 2020.0.3 (Biomatters Inc., Auckland, New Zealand).

### 2.5 DNA extraction from cacao beans

Microbial DNA was extracted from cacao beans following the method described in previous studies (Ahmadi et al., 2018) with minor modifications.

Briefly, 30 g of each sample were homogenized in 90 mL of saline solution (0.85% NaCl w/v) as cell suspension buffer (Verce et al., 2021). Suspensions were manually mixed in sterile sampling bags for 10 min. Then, 50 mL of the mixture were centrifuged at 500 × *g* for 10 min at room temperature (Thermo Scientific refrigerated centrifuge, GmbH am Kalkberg, Germany) to remove cacao debris. The supernatant was collected in a Falcon tube and the cell mass was obtained by centrifugation at 5,000 × *g* for 20 min at room temperature. After discarding the supernatant, the remaining pellet was resuspended in 500 μL of suspension buffer (10 mM Tris-HCl, 1 mM EDTA, 30 μL proteinase K at 20 mg/mL) in 2 mL microcentrifuge tubes, vortexed briefly, and incubated at 37 °C for 30 min. Next, 500 µL of lysis buffer (100 mM Tris-HCl; 50 mM EDTA; 0.5 M NaCl; 4% SDS; 2% polyvinylpolypyrrolidone (PVP)) were added to the cell mass and incubated at 70 °C for 30 min, with frequent mixing every 5 min, followed by the addition of 250 µL of potassium acetate (5 M, pH = 5.5). An equal volume of phenol/chloroform was then added to each tube and mixed by inversion. The upper aqueous phase containing the DNA was collected after centrifugation at 10,000 × g for 10 min at 4 °C.

The extracted DNA was precipitated by adding 600 μL of isopropanol and incubated for 5 min at room temperature. DNA was pelleted by centrifugation at 14,000 × *g* for 10 min at 4 °C (Thermo Scientific refrigerated centrifuge, GmbH am Kalkberg, Germany). Nuclease-free water was used as a negative control and processed identically to biological samples through DNA extraction and library preparation.

The purity, quality, and integrity of the extracted DNA was assessed by agarose gel electrophoresis, spectrophotometry (NanoDrop; Thermo Fisher Scientific), and fluorometry (Qubit; Thermo Fisher Scientific, Carlsbad, CA, United States).

### 2.6 Illumina shotgun sequencing and data processing

DNA from samples at each fermentation time point were submitted to a commercial laboratory for library preparation and shotgun sequencing on an Illumina MiSeq platform.

Raw sequences data were analyzed using the Omicsbox software (BioBam, Valencia, Spain). In this program, the obtained sequences were subjected to quality controls using the FASTQC tool (Andrews, 2010) then preprocessed by means of FASTQ using the Trimmomatic software (Bolger et al., 2014) to remove adapters and low-quality reads. The taxonomic composition of the samples was elucidated using the Kraken2 classifier which references the NCBI taxonomy database (Wood et al., 2019). Sequence data were normalized to the total number of counts as suggested elsewhere (Chen et al., 2021).

The resulting taxonomic matrix data was used to plot heatmaps of the taxa with ≥2% relative abundance at the order, family, genus, and species level in the *pheatmap* package 1.0.12 in R 4.2.3. This filtering threshold was chosen to enhance the reliability and interpretability of the visualization by focusing on the most dominant community members and reducing noise from low-abundance, potentially spurious reads (Nikodemova et al., 2023; Brunet et al., 2025).

### 2.7 Nanopore sequencing and data processing

The Rapid Barcoding Kit (SQK-RBK004) developed by Oxford Nanopore Technologies was employed for library preparation. For each sample, 200 ng of DNA were adjusted to a final volume of 7.5 μL with Nuclease-free water. DNA was then mixed with 2.5 µL of Fragmentation Mix RB01-12. The mixture was incubated at 30 °C for 1 min, followed by 80 °C for 1 min. Subsequently, 1 µL of rapid adapters (RAP) were incorporated into 10 µL of barcoded DNA and incubated at room temperature for 5 min. The prepared libraries were loaded onto a primed R9.4.1 flow cell and sequenced using the MinION device (Oxford Nanopore Technologies, United Kingdom).

MinION raw sequencing reads were basecalled using the MinKNOW software (ONT). Adapters were removed from basecalled sequences by means of Porechop 0.2.4 (Wick, 2017) and then uploaded to the cloud based EPI2ME fastq WIMP (What’s in my pot) workflow provided by Nanopore. Through this online resource, basecalled sequences were taxonomically assigned against a predesigned database based on NCBI taxonomy and the RefSeq database. Here, each read was classified based on the percent coverage and identity.

Data were normalized to the total number of counts and added to the taxonomic matrix obtained from Illumina shotgun sequencing. Then, the data was used to generate heatmaps of taxa with ≥2% relative abundance at the order, family, genus, and species level by utilizing the *pheatmap* function in R.

### 2.8 Hybrid assembly and functional annotation

Unicycler 0.5.0 (Wick et al., 2017) was utilized to perform hybrid assembly of Illumina and MinION sequencing data by using the default parameters of this tool. Assembly quality was evaluated using Quast 5.2.0 (Mikheenko et al., 2018). Subsequently, the assembled contigs were aligned with the NCBI non-redundant database by means of Diamond 2.0.15 (Buchfink et al., 2014).

The resulting alignments were processed using the *daa-meganizer*, a tool available within the MEGAN6 suite (Eberhard Karls Universität, Tübingen, Germany) (Beier et al., 2017), to enable taxonomic assignment based on the Lowest Common Ancestor (LCA) algorithm. Functional assignments were derived using MEGAN’s default mapping file, which links DIAMOND alignments to KEGG Orthology (KO) terms, facilitating the reconstruction of metabolic pathways relevant to cacao fermentation.

### 2.9 Statistical analysis

Spearman’s correlation analysis was used to compare the microbial communities of the samples as revealed by the different sequencing platforms. The correlation between the different platforms was considered very strong if the (rho) coefficient was ± 0.9 to 1, strong if it was ± 0.7 to 0.9, moderate if it was ± 0.5 to 0.7, weak if it was ± 0.3 to 0.5, or negligible if it was ± 0.0 to 0.3 (Mukaka, 2012; Nygaard et al., 2020).

Alpha diversity indices were calculated in the software Past 4.03 (Natural History Museum, Oslo, Norway). R was used to plot correlation graphs and alpha diversity indices by means of the *ggplot2* package 3.4.1.

Additionally, permutational multivariate analysis of variance (PERMANOVA) based on Bray–Curtis dissimilarities was performed with the *adonis2* function within the *vegan* package 2.6-4 to evaluate the effects of the sequencing platforms and the differences between fermentation time point samples at the species level.

## 3 Results

### 3.1 Overall NGS results

We first performed shotgun sequencing using Illumina and Nanopore platforms to profile microbial communities during cacao fermentation.

The profile of microbial communities during cacao fermentation were sequenced using both Illumina and Nanopore platforms. Shotgun sequencing on the Illumina MiSeq platform generated an average of 2,061,428 reads per sample, with an average of 1,017,916 classified reads representing 49.37% of the total reads filtered after QC (Table 1).

**TABLE 1:** Filtered reads after quality control and taxonomically classified reads, generated from Illumina and nanopore sequencing of cacao samples from different fermentation time points.

Sample information	Illumina			Nanopore		
Fermentation time (h)	Filtered reads	Classified reads	% of classified reads	Filtered reads	Classified reads	% of classified reads
0	1781413	550026	30.87	824	594	72.08
24	2398094	970555	40.47	456	331	72.58
48	1856044	718,155	38.69	3327	2,385	71.68
72	2322132	1655704	71.30	536	402	75
96	1949456	1195142	61.30	538	416	77.32
Mean	2061428	1017916	49.37	1,136	826	72.71

Quality control analysis revealed a median Phred score consistently above Q30 across the read length. Nanopore sequencing yielded an average of 1,136 reads per sample, with an average of 826 classified reads representing 72.71% of the total filtered reads after quality control of the basecalled sequences. Nanopore reads had a mean Phred-like quality score of 10.9 (median 10.0). The negative control produced only negligible read counts, confirming minimal background contamination.

### 3.2 Microbial taxa in fermented cacao samples revealed by Illumina and Nanopore sequencing

Next, we examined how microbial communities changed over time by analyzing taxonomic profiles from both sequencing platforms.

**FIGURE 1:**
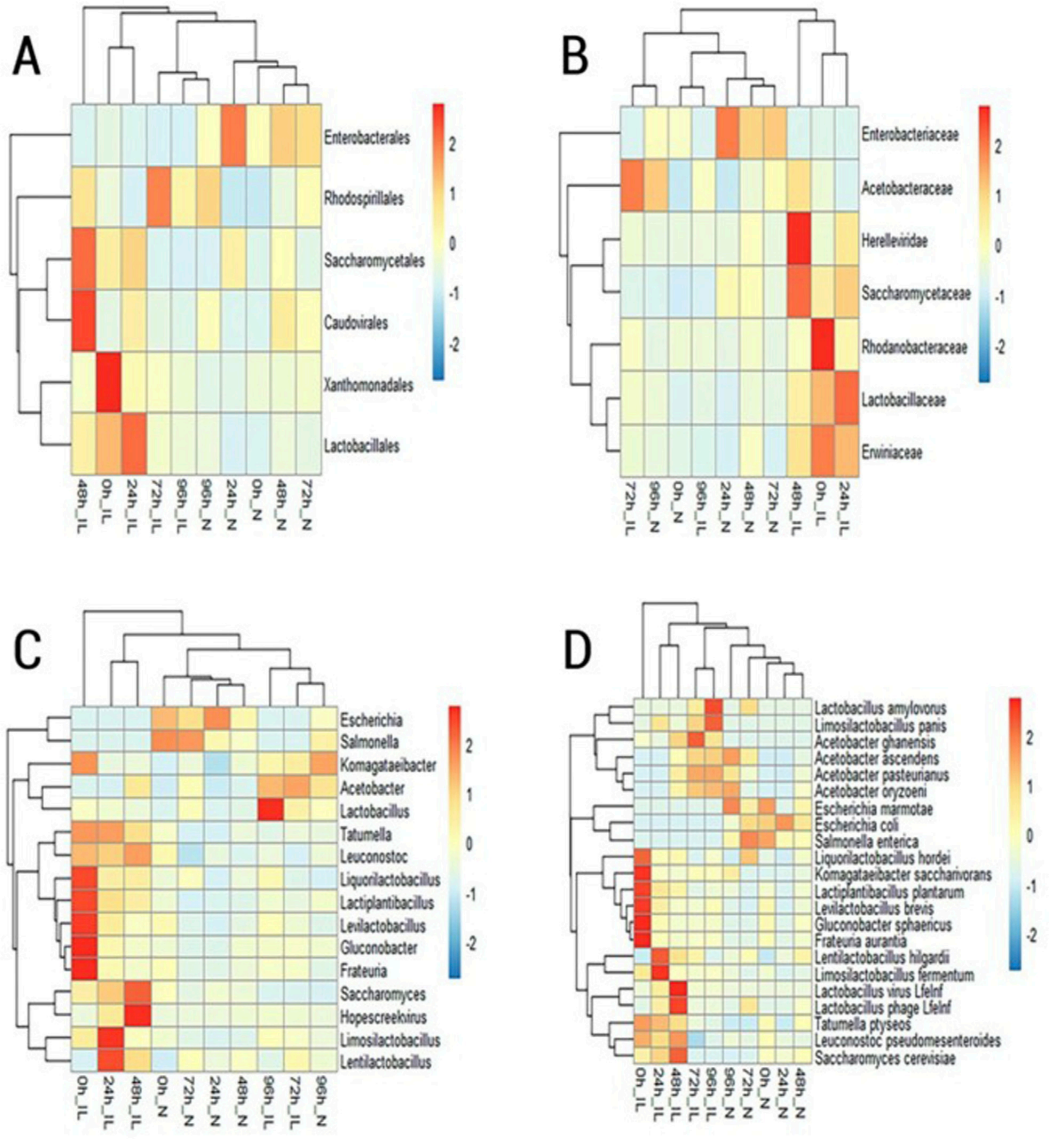
Heatmap of microbial taxa with ≥2% relative abundance identified by IL (Illumina) and N (Nanopore) sequencing at the **(A)** order, **(B)** family, **(C)** genus and **(D)** species level.

**FIGURE 2:**
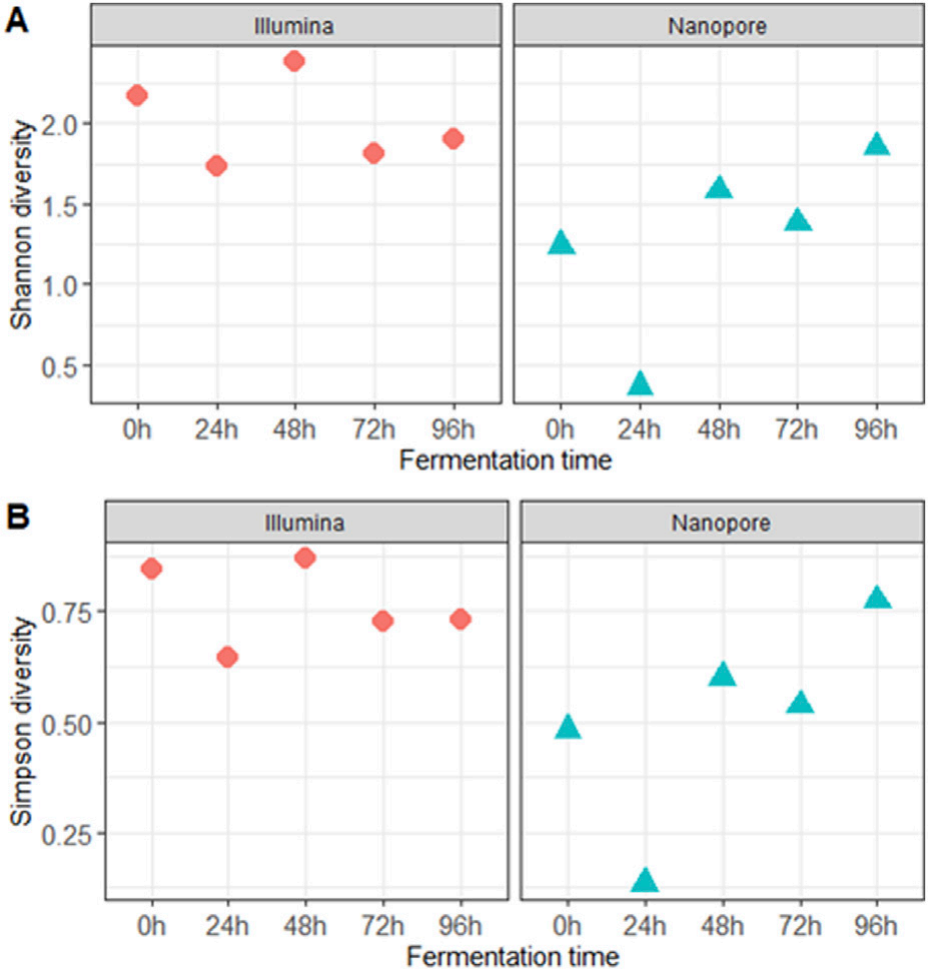
Alpha diversity indicators: **(A)** Shannon and **(B)** Simpson diversity indexes at the species level during the different fermentation times.

**FIGURE 3:**
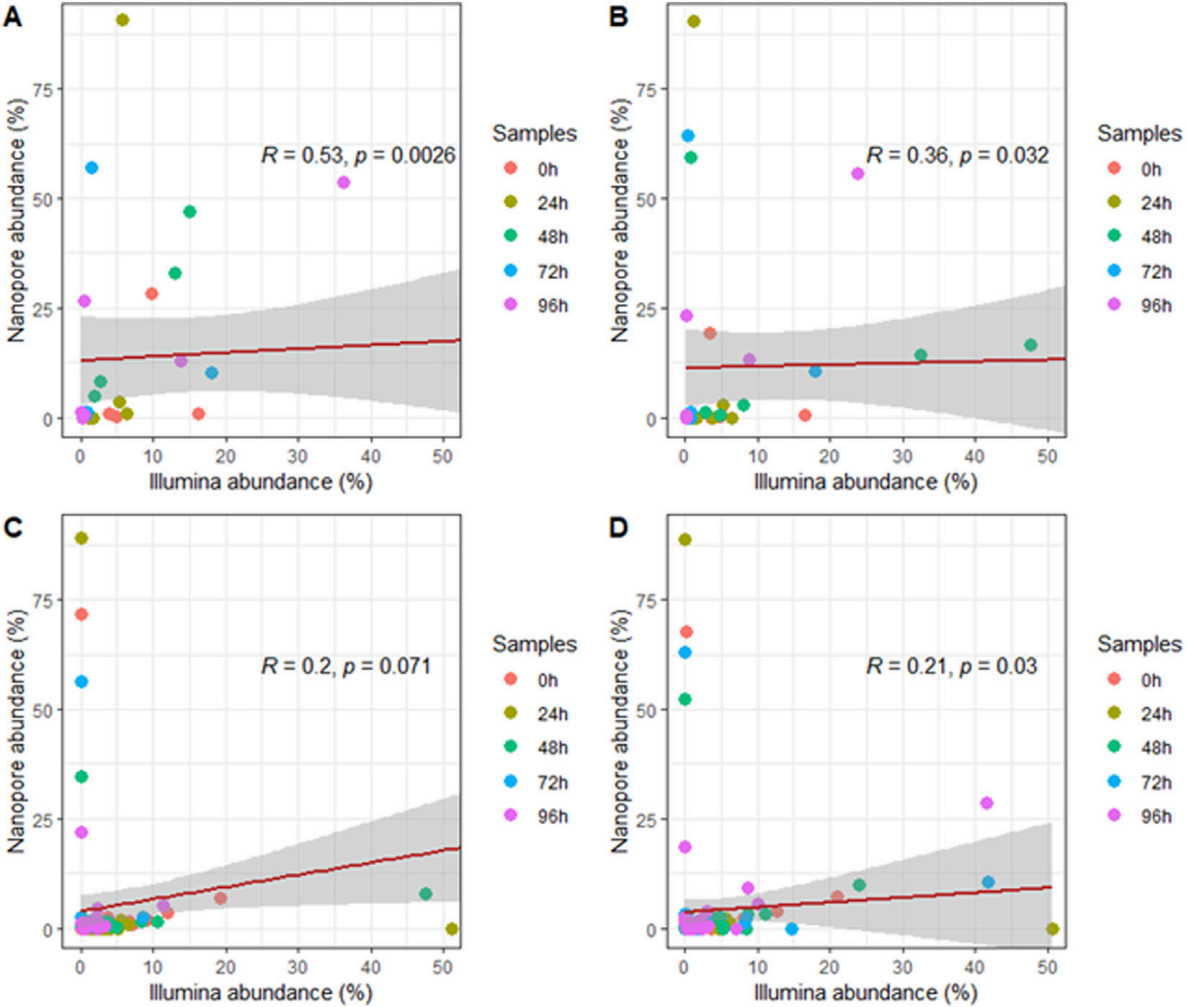
Correlation of microbial taxa identified at the level of **(A)** order, **(B)** family, **(C)** genus and **(D)** species in both sequencing platforms for all fermentation time point samples.

**FIGURE 4:**
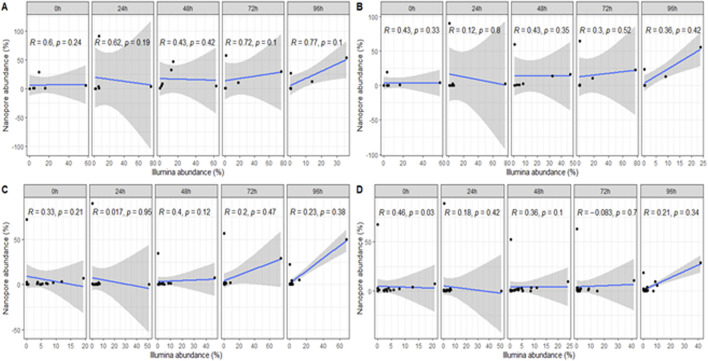
Correlation of identified microbial taxa at the **(A)** order, **(B)** family, **(C)** genus and **(D)** species level between sequencing platforms and individual samples.

Sequencing reads generated by the Illumina MiSeq platform and Nanopore sequencer were mapped against the NCBI database, as well as whole genomes in the RefSeq database for the bacterial, fungi and viral domains. The microbial classifications obtained were compared at the order, family, genus, and species level for each fermentation time point. Taxa with ≥2% relative abundance determined at the order, family, genus, and species level are displayed in heatmaps in Figures 1–4, respectively.

Illumina sequencing revealed a high abundance of the orders Caudovirales, Xanthomonadales Saccaromycetales, and Lactobacillales in the first 48 h of cacao fermentation. In the subsequent stages of the fermentative process (72 h and 96 h) elevated levels of the bacterial order Rhodospirillales were evidenced (Figure 1A). On the other hand, at the beginning of cacao fermentation (0 h, 24 h and 48 h); MinION sequencing, revealed a greater abundance of Enterobacterales and Saccharomycetales. At the end of the fermentation process (72 h and 96 h) a high detection of Rhodospirillales and Caudovirales was evidenced.

At the family level, analysis of the sequences generated by the Illumina MiSeq platform revealed a prevalence of the Saccharomycetaceae, Lactobacillaceae and Erwiniaceae in the samples corresponding to 0 h, 24 h and 48 h after the start of spontaneous fermentation. This trend later changed in the samples recollected at 72 h and 96 h of fermentation, where the highest abundance was recorded for the Acetobacteraceae family (Figure 1B). The analysis of Nanopore sequencing reads showed a high relative abundance of Enterobacteriaceae, and Saccharomycetaceae during the first 48 h of the fermentation process. Additionally, in the samples corresponding to 72 h and 96 h after the start of fermentation, Acetobacteraceae was one of the most frequently detected taxa followed by the Enterobacteriaceae family.

At the genus level, Illumina sequencing revealed high diversity at 0h, with lactic acid bacteria (*Levilactobacillus*, *Lactiplantibacillus*, *Liquorilactobacillus*), acetic acid bacteria (*Gluconobacter*), and environmental taxa (*Frauteria*) prevailing. At 24 h, *Limosilactobacillus* and *Lentilactobacillus* dominated, while at 48 h, *Saccharomyces* and *Hopescreekvirus* were most abundant. By 72 h–96 h, *Lactobacillus* and *Acetobacter* prevailed. Nanopore sequencing, in contrast, showed a dominance of *Escherichia* and *Salmonella* from 0 h to 48 h, with an increase in *Lentilactobacillus* at 48 h (Figure 1C). At 72 h, *Escherichia* and *Salmonella* remained abundant, along with *Acetobacter* and *Liquorilactobacillus*. At 96 h, *Acetobacte*r and *Komagataeibacter* were predominant.

At the species level, Illumina reads at 0 h showed *Levilactobacillus brevis*, *Lantiplantibacillus plantarum*, *Liquorilactobacillus hordei*, *Gluconobacter sphaericus*, and *Frauteria aurantia*. At 24 h, *Limosilactobacillus fermentum* and *Lentilactobacillus hilgardii* were most abundant. By 48 h, *Saccharomyces cerevisiae* and phages *Lactobacillus phage Lfelnf* and *Lactobacillus virus Lfelnf* dominated. *Acetobacter ghanensis* was prevalent at 72 h, and *Limosilactobacillus panis* and *Lactobacillus amylovorus* increased at 96 h. Nanopore sequencing revealed *E. marmotae* and *Salmonella enterica* at 0 h–48 h, with *Escherichia coli* peaking at 24 h (Figure 1D). At 72 h, *Liquorilactobacillus hordei, S. enterica*, and *E. coli* were dominant. By 96 h, *Acetobacter ascendens*, *Acetobacter oryzoeni*, and *Escherichia marmotae* prevailed.

The alpha diversity analysis revealed differences in species count, with Illumina sequencing yielding higher diversity values than Nanopore during the first 72 h of fermentation. Both sequencing methods showed the lowest diversity values in samples fermented for 24 h, while the highest diversity was observed after 48 h using Illumina and after 96 h using Nanopore (Figures 2A,B).

PERMANOVA analysis was conducted to assess microbial profiles at the species level. The results showed that the sequencing technique accounted for 19% of the total variations at this taxonomic level, while fermentation time contributed 37% of the variation (Supplementary Table S1).

Fermentation success was confirmed through evaluation by a trained sensory panel. All samples included in the study were verified to have undergone successful fermentation based on sensory attributes; however, detailed sensory profiling was beyond the scope of this study.

### 3.3 Illumina shotgun and nanopore sequencing correlation

To compare the performance of both methods, we conducted a correlation analysis of taxonomic assignments generated by each platform. This comparison aimed to assess the degree of consistency in microbial community profiles derived from the two approaches. Spearman analysis revealed a significant correlation between the data obtained by both platforms at the order, family, and species levels (Figures 3A,B,D). However, the correlation at the genus level was not significant (Figure 3C).

The analysis of individual fermentation time point samples between the sequencing technologies showed moderate and weak positive correlations between the sequencing platforms at the order and family level for most of the samples. Nonetheless, correlations at the genus and species level were negligible. Despite this, no significant correlation was evidenced at any taxonomic level (Figures 4A–D).

**FIGURE 5:**
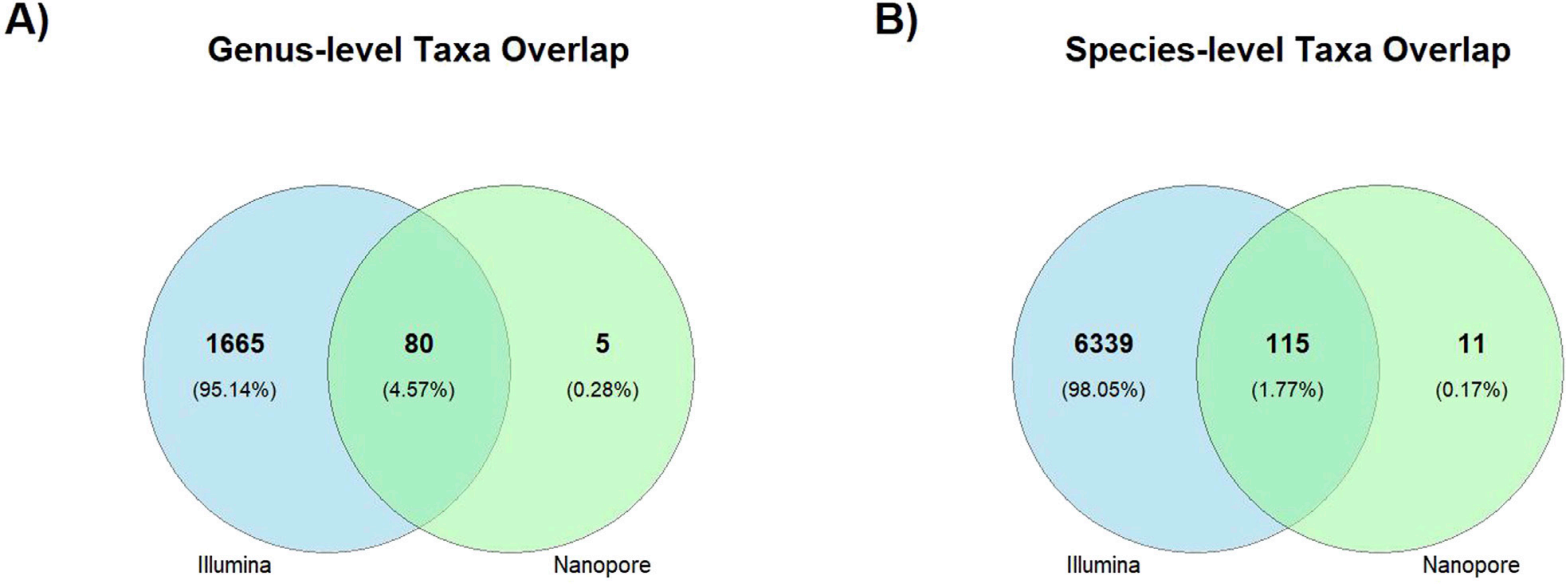
Venn diagrams showing taxonomic overlaps between Illumina and Nanopore sequencing platforms. The diagrams display the number and percentage of taxa uniquely and jointly identified at the genus **(A)** and species **(B)** levels.

Correlation results are consistent with the number of shared taxa displayed on (Table 2) that were detected at the different taxonomic levels by Illumina and Nanopore sequencing platforms. Figure 5 illustrates this overlap through Venn diagrams, showing the number and percentage of taxa shared and unique to each platform at the genus (panel A) and species (panel B) levels. At the species level, only 115 taxa representing 1.77% of the total identified taxa were detected by both sequencing approaches.

**TABLE 2:** Number of taxa identified at the different taxonomic levels by Illumina and Nanopore sequencing.

Taxonomic level	Total	Illumina only	Shared	Nanopore only
Order	235	214 (91.06%)	19 (8.08%)	2 (0.93%)
Family	487	437 (89.73%)	49 (10.06%)	1 (0.20%)
Genera	1750	1665 (95.14%)	80 (4.57%)	5 (0.28%)
Species	6465	6339 (98.05%)	115 (1.77%)	11 (0.17%)

### 3.4 Microbial species identified by Sanger sequencing

To complement the microbial profiles obtained through NGS, we employed culture-based isolation followed by Sanger sequencing. This approach enabled the recovery of viable microorganisms and provided an independent confirmation of taxonomic identities, thereby strengthening the reliability of the NGS-based community analysis. A total of 249 microbial isolates were obtained, including 158 isolates on MRS, 50 on GYC, and 41 on PDA, from which 17 unique species were identified in 34 species-fermentation-time combinations. Table 3 shows the species that share at least 98% identity with the DNA sequences of the individual isolates. Results show that all the species detected by culture-based methods were also detected by Illumina’s NGS but only 19 species-fermentation-time combinations (55%) were detected using nanopore sequencing.

**TABLE 3:** Species identified by Sanger sequencing of microbial isolates; N represents (Nanopore) and IL (llumina) sequencing.

Fermentation time point (h)	Sanger sequencing	NGS detection
0	*S*. *cerevisiae*	N, IL
	*L*. *pseudomesenteroides*	N, IL
	*L*. *plantarum*	N, IL
	*Lactiplantibacillus pentosus*	IL
	*L*. *brevis*	N, IL
	*Priestia megaterium*	IL
	*Bacillus cereus*	IL
	*Bacillus pumilus*	IL
	*Bacillus altitudinis*	IL
	*Staphylococcus capitis*	IL
24	*S*. *cerevisiae*	N, IL
	*L*. *plantarum*	N, IL
	*L*. *pseudomesenteroides*	N, IL
	*Bacillus altitudinis*	IL
	*Bacillus cereus*	IL
48	*S*. *cerevisiae*	N, IL
	*L*. *plantarum*	N, IL
	*L*. *brevis*	N, IL
	*Staphylococcus saprophyticus*	IL
	*Staphylococcus epidermidis*	IL
	*L*. *hilgardii*	N, IL
	*L*. *mali*	N
	*Bacillus cereus*	N
72	*L*. *plantarum*	N, IL
	*L. brevis*	N, IL
	*L*. *pseudomesenteroides*	N, IL
	*Leuconostoc mesenteroides*	IL
	*Lacticaseibacillus paracasei*	IL
	*Staphylococcus epidermidis*	IL
96	*L*. *plantarum*	N, IL
	*L*. *mali*	IL
	*L*. *brevis*	N, IL
	*A*. *pasteurianus*	N, IL
	*Staphylococcus epidermidis*	IL

Overall, Sanger sequencing of microbial isolates allowed the identification of lactic acid bacteria, acetic acid bacteria, and yeasts. The three sequencing methods allowed the detection of the main species of yeast (*S.cerevisiae*), lactic acid bacteria (*L.plantarum*, *L. pseudomesenteroides*, *L. brevis*, *L. hilgardii, L. mali*) and acetic acid bacteria (*Acetobacter pasteurianus*).

### 3.5 Metabolism of the fine flavor cacao fermentation microbiome

To explore microbial function, we used a hybrid Illumina-Nanopore assembly for metabolic pathway analysis.

**FIGURE 6:**
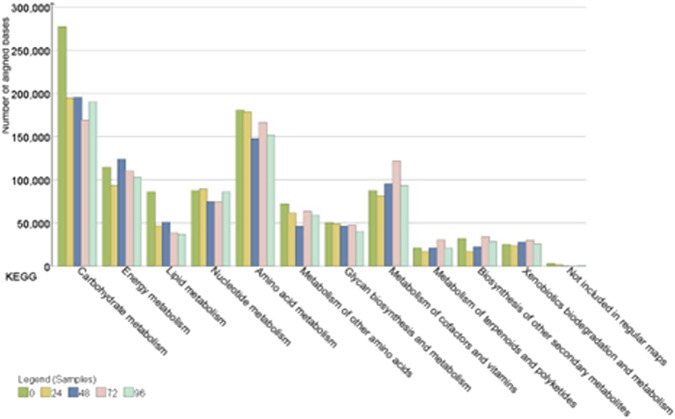
KEGG genes encoding different metabolic pathways in the samples from 0, 24, 48, 72 and 96 h of fermentation.

The hybrid assembly statistics are detailed in (Supplementary Table S2). The functional annotation of the hybrid assembly of Nanopore and Illumina data showed that KEGG pathways mapped to the metabolism of carbohydrates and amino acids were the most abundant throughout the fermentation process (Figure 6). The main orthologous genes, enzymes, enzyme codes, number of assigned reads and distribution of microbial genera during the cacao fermentation process are shown in (Table 4).

**TABLE 4:** Main metabolic pathways, enzymes, orthologous genes, and their microbial distribution in fermented cocoa samples.

Pathway	Enzyme name	Enzyme EC number	KEGG orthology	Read count	Fermentation time (h)	Distribution of microbes
Penthose phosphate	Fructose-1,6-bisphosphatase III	3.1.3.11	K04041	13,410	0	*Liquorilactobacillus, Lactiplantibacillus*
				7,540	48	*Liquorilactobacillus*
	Transketolase	2.2.1.1	K00615	8264	0	*Paucilactobacillus, Levilactobacillus, Limosilactobacillus*
				2,733	24	*Limosilactobacillus*
Pyruvate metabolism	Acetyl-CoA carboxylase, biotin carboxylase subunit	6.4.1.2 6.3.4.14	K01961	7,886	0	*Paucilactobacillus, L*. *fermentum*
				4,596	24	*Liquorilactobacillus*
				6394	48	*Limosilactobacillus, Acetobacter*
				4,347	72	*Limosilactobacillus, Acetobacter*
	Pyruvate carboxylase	6.4.1.1	K01958	6476	0	*Paucilactobacillus, Liquorilactobacillus*
				7,587	48	*Liquorilactobacillus*
				4,761	96	*Liquorilactobacillus*
Glycolysis, Gluconeogenesis	Phosphoglucomutase	5.4.2.2	K01835	4,266	96	*Acetobacter, Limosilactobacillus, Komagataeibacter*
	Pyruvate decarboxylase	4.1.1.1	K01568	2,158	96	*Acetobacter*
Alanine, aspartate and glutamate metabolism	4-aminobutyrate aminotransferase	2.6.1.19	K00823	5,176	0	*Limosilactobacillus, Paucilactobacillus*
				5,622	24	*Liquorilactobacillus*
	Succinate-semialdehyde dehydrogenase/glutarate-semialdehyde dehydrogenase	1.2.1.16 1.2.1.79 1.2.1.20	K00135	9036	24	*Lentilactobacillus, Secundilactobacillus, Liquorilactobacillus, Limosilactobacillus*
				4,592	72	*Acetobacter, Komagataeibacter, Limosilactobacillus*
Cysteine and methionine metabolism	Aspartate aminotransferase	2.6.1.1	K00812	4,070	72	*Acetobacter, Limosilactobacillus*
	Glutamate-cysteine ligase	6.3.2.2	K01919	6833	96	*Limosilactobacillus, Acetobacter*
Biosynthesis of various plant secondary metabolites	Beta-glucosidase	3.2.1.21	K05349	5,350	0	*Paucilactobacillus, Limosilactobacillus*
Caffeine metabolism	Methylxanthine N3-demethylase	1.14.13.179	K21723	1907	72	Acetobacteraceae*, Paracoccus*
	Methylxanthine demethylase	1.14.13.128	K21724 7	3385	48	Acetobacteraceae*, Paracoccus*
				2,437	96	Acetobacteraceae*, Paracoccus*
Flavonoid biosynthesis	Anthocyanin reductase	1.3.1.77	K08695	332	96	*Levilactobacillus*

#### 3.5.1 Carbohydrate metabolism

The carbohydrate metabolism was one of the most prevalent metabolic pathways within the Nacional x Trinitario cacao fermentation microbiota.

In detail, the genes that encode pyruvate and pentose phosphate metabolism were the most detected during the first 48 h of cocoa fermentation. At this stage of the fermentative process, it was observed that the genes coding the enzyme fructose-1,6-bisphosphatase (EC: 3.1.3.11, 10475 reads), as well as transketolase (EC: 2.2.1.1, 5498.5 reads) were the most abundant within the pentose phosphate metabolic pathway and were attributed to lactic acid bacteria of the genera *Paucilactobacillus*, *Limosilactobacillus* and *Lactiplantibacillus*. Additionally, at the beginning of fermentation, the enzyme acetyl-CoA carboxylase, biotin carboxylase subunit (EC: 6.4.1.2 6.3.4.14, 5692.66 reads) was the most predominant within the metabolic pathway of pyruvate and were attributed to lactic acid bacteria of the genera *Liquorilactobacillus* and *Limosilactobacillus*, as well as acetic acid bacteria of the genus *Acetobacte*r. The enzyme pyruvate carboxylase (EC: 6.4.1.1, 7031 average reads) was detected at 0 and 48 h of fermentation and was assigned to the genera *Acetobacter*, *Komagataeibacter* and *Limosilactobacillus*.

After 48 h of fermentation, the enzyme acetyl-CoA carboxylase, biotin carboxylase subunit, continued to be the most abundant, followed by KEGG genes coding the metabolic pathway of glycolysis and gluconeogenesis including phosphoglucomutase (EC: 5.4.2.2, 4266 reads) attributed to bacteria of the genus *Limosilactobacillus* and pyruvate decarboxylase (EC: 4.1.1.1, 2158 reads), which was related to acetic acid bacteria of the genera *Acetobacter* and *Komagataeibacte*r.

#### 3.5.2 Amino acid metabolism

Amino acid metabolism was one of the most active pathways during cacao fermentation.

Results evidenced the abundant presence of genes related to the biosynthesis of valine, leucine, and isoleucine and alanine, aspartate, and glutamate during the first 48 h of fermentation. In this sense, genes coding the enzyme 4-aminobutyrate aminotransferase (EC: 2.6.1.19, 5399 reads) attributed to the genera *Liquorilatobacillus* were detected. Additionally, in this stage of the fermentation process, the presence of genes coding the enzyme succinate-semialdehyde dehydrogenase (EC: 1.2.1.16 1.2.1.79, 9036 reads) attributed to the genera *Lentilactobacillus*, *Liquorilactobacillus* and *Limosilactobacillus* were observed.

In the subsequent hours of fermentation, the metabolic pathway of alanine, aspartate, and glutamate continued to be one of the most prevalent, followed by the cysteine and methionine metabolism. In this regard, genes coding the aspartate aminotransferase enzyme were observed (EC: 2.6.1.1, 4070 average reads) and were attributed to the *Acetobacter* and *Limosilactobacillus* genera.

#### 3.5.3 Biosynthesis of secondary metabolites

Finally, we monitored the progression of secondary metabolite biosynthesis throughout fermentation.

Functional analysis showed the presence of the genes coding for the metabolism of various secondary metabolites at the onset of the fermentation process. In detail, genes encoding the beta-glucosidase enzyme were identified (EC: 3.2.1.21, 5350 reads), and were attributed to the metabolism of lactic acid bacteria genera including *Paucilactobacillus* and *Limosilactobacillus*.

An interesting finding was observed at 48 h after the start of fermentation, where coding genes of enzymatic reactions for the biosynthesis of theobromine were observed and were related to the metabolic activity of Acetobacteraceae and *Paracoccus* (EC:1.14.13.179, 3768 reads). In the final stage of fermentation, the presence of genes that encode the anthocyanin reductase enzyme (EC: 1.3.1.77, 332 reads) attributed to the genus *Levilactobacillus* within the flavonoid biosynthesis pathway were detected.

## 4 Discussion

In the current study, we characterized the microbial communities present during Nacional x Trinitario cacao bean fermentation. For this purpose, three different approaches based on Illumina and Nanopore metagenomic sequencing, as well as Sanger sequencing of individual isolates, were used for characterizing the microbial composition of samples taken at 0 h, 24 h, 48 h, 72 h, and 96 h after the start of the spontaneous fermentation process. Additionally, the role of the microbiota in the fine flavor cacao fermentation process was investigated by performing a functional annotation of the hybrid assembly of Nanopore and Illumina sequencing data.

Culture-dependent assessment followed by Sanger sequencing revealed the presence of several LAB, AAB, yeasts, and spore-forming bacteria (e.g., *Bacillus*) at the start of fermentation. As fermentation progressed, microbial dynamics became dominated by LAB, followed by AAB. These findings are consistent with previous studies on cacao fermentation (Camu et al., 2007; Nielsen et al., 2007; Camu et al., 2008; Constante Catuto et al., 2024; Camu et al., 2007; 2008; Nielsen et al., 2007; Constante Catuto et al., 2024). Both NGS and Sanger sequencing were consistent in the detection of *L. plantarum*, *L. pseudomesenteroides*, *L. brevis*, *L. hilgardii* and *L. mali*. According to Papalexandratou et al. (2011b) and Vuyst and Weckx (2015), several LAB species can utilize citrate as an energy source, thereby shortening the fermentation period and producing flavor precursors *via* pyruvate metabolism.

*Saccharomyces cerevisiae* was detected at the onset (0 h–48 h) of fermentation by all three sequencing methods. This yeast’s metabolic activity is closely linked to carbohydrate metabolism, a major pathway contributing to flavor development in cocoa fermentation (Almeida et al., 2020; Santander Muñoz et al., 2020).

At the end of the fermentation process, *A*. *pasteurianus* was consistently identified as the dominant AAB species by all methods, in line with prior reports (Lefeber et al., 2011; Soumahoro et al., 2020). Conversely, viral taxa such as *L. phage Lfelnf* and *L. virus Lfelnf* were detected only by Illumina and Nanopore sequencing. These viruses are known to infect *L*. *fermentum* (Liu et al., 2015; Tochilina et al., 2019), and their presence in cacao fermentation has only been reported in a few studies (Illeghems et al., 2015; Agyirifo et al., 2019; Almeida et al., 2020). Agyirifo et al. (2019) proposed that bacteriophages may positively influence fermentation by lysing LAB cells and releasing enzymes that catalyze the formation of aroma compounds. The detection of these viral taxa adds to the growing but still limited evidence that phages may play an active and underexplored role in shaping microbial dynamics during cacao fermentation.

Additionally, NGS uniquely detected a high abundance of *A*. *ascendens* in the final fermentation stage (72 h–96 h). This species has rarely been associated with cacao fermentation, with only one report from Brazil involving Forastero and Trinitario beans (Carolina et al., 2021). Its consistent presence at the late stage of fermentation in our samples suggests it may have a more relevant role in the microbial succession of cacao fermentations than previously recognized. While further studies are needed to elucidate its functional contribution, this finding broadens the known diversity of AABs involved in the process. Together, these observations highlight several rarely described microorganisms in cacao fermentation such as specific bacteriophages and *A. ascendens* which enhance the novelty and significance of our findings.

It is also important to recognize that Sanger sequencing and NGS are fundamentally different technologies, producing distinct data types. As such, direct comparison is challenging and often requires generating consensus sequences from Nanopore and Illumina reads (Paul et al., 2018; Sahlin et al., 2021; Marshall et al., 2023).

Our findings indicate that throughput in Nanopore sequencing was limited compared to Illumina. Although Nanopore provided valuable insights into the microbial dynamics of cacao fermentation, the relatively low number of reads per sample (average of 1,136) represents a notable limitation. This likely stems from the challenging nature of fermented cacao matrices, which contain polysaccharides, polyphenols, and other inhibitory compounds that interfere with DNA extraction and library preparation quality (Ramos et al., 2014). Despite using the rapid sequencing kit (SQK-RBK004), its high DNA input requirement (400 ng) further constrained sequencing performance. Additionally, Nanopore reads exhibited a mean Phred-like quality score of ∼10.9, reflecting the known higher error rates compared to Illumina. This lower accuracy may reduce species-level assignment precision, particularly for low-abundance taxa (Delahaye and Nicolas, 2021).

While dominant groups such as *S. cerevisiae*, *L. plantarum*, and *A. pasteurianus* were generally detected across all methods, discrepancies were noted in early fermentation stages. Nanopore showed limited detection of LAB at the onset of fermentation, reflecting differences in sensitivity and taxonomic resolution. These inconsistencies highlight the importance of cautious cross-platform interpretation. In this regard, future studies should consider improved DNA extraction methods, newer ONT kits requiring less input, and enhanced bioinformatic strategies such as updated basecallers like ONT’s Dorado and optimized EPI2ME workflows with stronger error correction to improve sequencing quality and accuracy (Buddle et al., 2024; Hong et al., 2024).

Illumina and Nanopore sequencing, analyzed against NCBI and RefSeq databases, revealed similar structural changes in microbial communities over fermentation at order, family, genus and species level showing a prevalence of yeasts, LAB, and AAB, consistent with findings in Criollo, Forastero, and Trinitario fermentations (Camu et al., 2007; Agyirifo et al., 2019; Díaz-Muñoz et al., 2021).

Hierarchical clustering (Figure 1) revealed time-dependent grouping of samples across all taxonomic levels, indicating clear shifts in community structure during fermentation. Early stages (0 h–48 h) clustered separately from late stages (72 h–96 h), reflecting a temporal transition toward communities dominated by fewer, fermentation-adapted taxa. This pattern was consistent across both Illumina and Nanopore datasets.

Notably, Nanopore detected a higher abundance of Enterobacteriaceae throughout fermentation. This group may originate from soil or cacao plant tissues (Papalexandratou et al., 2011a; Almeida Câmara Leite et al., 2013), and similar patterns have been reported in fermented vegetables like Chinese spicy cabbage and Paocai (Cao et al., 2017; Liu et al., 2019). Both platforms showed strong agreement in taxonomic composition at the order and family levels across all time points, as confirmed by correlation and heatmap analysis, aligning with previous studies (Shin et al., 2016; Nygaard et al., 2020). Therefore, while either sequencing approach appears suitable for capturing broad microbial trends during fermentation, higher taxonomic resolution at the genus or species level may require complementary methods.

Differences in performance that resulted in dissimilar alpha diversity indicators between Nanopore and Illumina data were observed (Figure 2). These disparities in alpha diversity likely reflect the inherent sensitivity and limitations of each sequencing platform, Nanopore may miss or misclassify certain taxa due to relatively lower accuracy and throughput observed in this study, while Illumina may better resolve community richness but be constrained by read length (Van Uffelen et al., 2024). One limitation of this study is the limited number of biological replicates per time point, which may affect the resolution of microbial shifts.

Several factors are likely to contribute to discrepancies and weaker correlations (Figures 3 and 4) observed at finer taxonomic levels. First, DNA extraction from fermented cacao is notoriously difficult due to the presence of polyphenols and complex carbohydrates that inhibit enzymatic reactions (Schrader et al., 2012). Such matrix effects may differentially impact extraction efficiency for high-molecular-weight DNA, favoring one platform over another. Second, biases in sequencing chemistry, such as Nanopore’s relatively high error rates or Illumina’s shorter read lengths can influence taxonomic classification accuracy (Stevens et al., 2023).

Third, the use of different bioinformatics pipelines adds further variability. The ONT cloud-based workflow fastq What’s in My Pot (WIMP), as noted by Brown et al. (2017), although suitable for rapid taxonomic profiling, offers less flexibility for metagenomic analysis due to constraints such as limited database customization and fewer options for algorithmic fine-tuning, which can impact taxonomic resolution and quantification accuracy. In contrast, classifiers like Kraken2 allow more customizable and comprehensive reference databases, improving detection and relative quantification of microbial taxa in complex communities. These pipeline-specific differences can result under or overestimation of specific taxa within a sample (Acharya et al., 2019; Winand et al., 2020).

Additionally, only 1.77% of species-level taxa were shared between Illumina and Nanopore platforms (Figure 5), underscoring the limitations of using a single sequencing approach for fine-resolution microbial profiling. This low overlap likely reflects platform specific biases that affect the detection of low-abundance or poorly annotated taxa (Stevens et al., 2023), which in turn may lead to an underrepresentation of microbial diversity and ecological interactions. Therefore, species-level outcomes should be interpreted with caution, and integrating complementary sequencing strategies may be necessary to achieve a more complete representation of microbial diversity.

Llumina and Nanopore sequencing showed that the initiation of fermentation was characterized by diverse microbial genera, including LAB (*Lactiplantibacillus*, *Levilactobacillus*, *Liquorilactobacillus*), AAB (*Gluconobacter*), and environmental or enterobacterial taxa like *Frateuria*, *Salmonella*, and *Escherichia*. This microbial diversity stems from the cacao pulp’s exposure to external sources such as pod surfaces, soil, machetes, workers’ hands, and fermentation containers (Viesser et al., 2021). At this stage, the abundance of genes associated with pyruvate and pentose phosphate metabolism suggests a constant carbon supply (Agyirifo et al., 2019). LAB showed homolactic metabolism, producing ethanol, acetate, lactate, and acetoin (Gänzle, 2015), and were also involved in fatty acid biosynthesis *via* acetyl-CoA carboxylase (Gurav and Bokade, 2010). Additionally, *Acetobacter, Komagataeibacter* and *Limosilactobacillus* converted pyruvate to oxaloacetate, a precursor of aspartate, through pyruvate carboxylase (De Vries, 2006).

Results showed that, as fermentation progressed other microbial groups started to predominate, for instance, Illumina sequencing revealed that yeasts (*Saccharomyces*) populations reached a peak at 48 h into fermentation. The presence of this yeast has been widely reported in the fermentation of different cocoa varieties across the world (Jespersen et al., 2005; Moreira et al., 2013; Batista et al., 2015). In this context, previous research (Dzialo et al., 2017) suggest that yeast metabolic activities cause a portion of the carbon getting transported to the Krebs cycle, which in turn allows the formation of aroma precursors by means of a series of biochemical reactions related to the amino acid metabolism. Furthermore, it is worth noting that the abundance of the viral genus *Hopescreekvirus* increased at this fermentation time point. This genus has one known species and was only recently reported by (Liu et al., 2015; Greiner et al., 2018; González-Orozco et al., 2023).

On the other hand, Nanopore sequencing revealed that enterobacterial genera were predominant over the course of fermentation. Within this microbial group, only a subset of genera and species was identified, namely, *E. coli, E. marmotae* and *S. enterica* (Figures 2A,B). Other researchers have also reported the involvement of enterobacteria in the cacao fermentation (Garcia-Armisen et al., 2010; Papalexandratou et al., 2011a; 2013; Hamdouche et al., 2015). Enterobacterial taxa are thought to contribute to glucose conversion into lactic acid and citric acid (Grimont and Grimont, 2006). Nonetheless, their persistent detection at high levels *via* Nanopore warrants critical interpretation. Rather than solely reflecting a dominant, metabolically active population, this finding may highlight a platform-specific bias. Several technical factors could contribute to this observation.

First, a reference database bias likely influenced the taxonomic classification. Taxa like *E. coli* and *Salmonella* are among the most sequenced organisms, with thousands of complete, high-quality reference genomes available in public databases (Brown et al., 2021; Horesh et al., 2021). Bioinformatics classifiers, when faced with the longer but higher error rate reads from Nanopore, may preferentially assign them to these “best-match” genomes over the less-complete or more fragmented genomes of niche fermentative taxa (Winand et al., 2020; Wick et al., 2023). This can create an illusion of high abundance for well-characterized organisms. Second, the DNA from these Gram-negative bacteria may be more efficiently extracted and amplified compared to that from thick-walled Gram-positive bacteria (LAB) and yeasts, further skewing their representation in the sequencing library (De Bruin et al., 2019).

The final stage of fermentation (72 h–96 h) as revealed by Nanopore and Illumina sequencing was characterized by an increment in the relative abundance of AABs including *Acetobacter* and *Komagataeibacter* which was consistent with findings from other cacao studies (Serra et al., 2019; Pacheco-Montealegre et al., 2020). The enzyme pyruvate decarboxylase (EC: 4.1.1.1) was attributed to the metabolic activities of these genera and it has been implicated in the decarboxylation of pyruvate to acetaldehyde, a volatile compound known to contribute to fruity aroma notes in fermented products (Peters et al., 2013).

It is worth noting that Illumina sequencing also revealed that *Lactobacillus* was abundant at 72 h after the start of the fermentation process. LAB are important actors of the fermentative process since they produce lactic acid that diffuses into the seed, which subsequently allows the activation of endogenous enzymes that contribute to the generation of the distinctive chocolate flavor and aroma (De Vuyst and Weckx, 2016; Viesser et al., 2021). Overall, the main genera identified in the present study are in accordance with previous research (Meersman et al., 2013; Bortolini et al., 2016; Carolina et al., 2021).

Amino acid metabolism (Figure 6) emerged as a central functional pathway in our data, strongly linked to the microbial production of volatile flavor compounds (Lima et al., 2022). Genes coding for various enzymes (EC: 2.6.1.19, EC: 1.2.1.16) attributed to various LAB genera that form succinate which then enters the tricarboxylic acid cycle (Sahab et al., 2020; Xia et al., 2022). Additionally, genes related to valine, leucine, and isoleucine metabolism were detected, supporting the microbial synthesis of flavor-active compounds like benzaldehyde and 2-phenylethanol, known for imparting fruity, malty, and floral notes (Pires et al., 2014; Díaz-Muñoz et al., 2021; Quelal et al., 2023).

Moreover, genes attributed to *Acetobacter* and *Limosilactobacillus* were associated with the enzyme aspartate aminotransferase, which participates in the biosynthesis of diacetyl and acetoin, volatile compounds that contribute buttery and creamy aromas to fermented cacao (Ardö, 2006; Tian et al., 2020). The detection of glutamate-cysteine ligase in these genera also suggests microbial involvement in the production of γ-glutamyl peptides, which enhance umami and overall flavor complexity (Xie and Gänzle, 2021).

Beyond aroma development, our functional annotation also highlighted pathways relevant to the health-promoting potential of cacao. Genes encoding β-glucosidase enzymes, mainly from *Paucilactobacillus* and *Limosilactobacillus* were found to catalyze the release of polyphenol aglycones, reducing astringency and bitterness while enhancing antioxidant availability (Rodríguez et al., 2004; Llano et al., 2025). These enzymes also break down cellulose into glucose monomers, supporting applications like bioethanol production (Harun and Danquah, 2011; Tan and Lee, 2014).

Lastly, genes involved in the biosynthesis of theobromine, a methylxanthine alkaloid with antioxidant and cardiovascular protective effects, were detected, further linking microbial activity to both sensory and nutritional quality (Brunetto et al., 2007; Jean-Marie et al., 2021; Pagliari et al., 2022).

To optimize the application of MinION sequencing in future cacao metagenomic studies, several improvements should be considered. First, selecting a DNA extraction method that yields high molecular weight and inhibitor-free DNA is critical for capturing the full microbial diversity of cacao fermentation. In this sense, incorporating purification techniques, such as AMPure XP bead-based cleanup is recommended, as it enhances DNA quality and optimizes subsequent sequencing outcomes (Angthong et al., 2020).

Second, the use of specialized enzymatic lysis cocktails can improve DNA recovery from hard-to-lyse taxa such as fungi and Gram-positive bacteria, which are often underrepresented (Langsiri et al., 2025). Third, leveraging ONT’s latest flow cells and updated library preparation kits, which support ultralong reads (∼100 kb) with higher basecalling accuracy, may improve taxonomic resolution (Sharma et al., 2025).

Finally, recent bioinformatics tools such as Dorado’s *dorado correct* (v0.9.1), which integrates the HERRO deep learning algorithm, offer effective raw read error correction, enhancing reliability of species-level assignments in complex communities (Vereecke et al., 2025). Integrating these advances can significantly improve data quality, depth, and interpretability in long-read metagenomics of cacao fermentation.

## Data Availability

The datasets generated for this study can be found in the SRA Database of NCBI: https://www.ncbi.nlm.nih.gov/sra/PRJNA1257864.
