# Supplementary material for: Multi-platform metagenomic characterization of the microbial community during spontaneous cacao fermentation
Source: Front Bioeng Biotechnol. 2025 Aug 26;13:1630515. doi: 10.3389/fbioe.2025.1630515 (PMC12417404; doi:10.3389/fbioe.2025.1630515)
Supplement: Supplementary file 1 [file Table1.docx]

Supplementary Material

Multi-platform metagenomic characterization of the microbial community during spontaneous cacao fermentation.

**Joel Tigrero Vaca, Mirian Villavicencio****-Vásquez, Jonathan Coronel, Juan M. Cevallos Cevallos ^*^**

*** Correspondence:** Juan M. Cevallos Cevallos: jmceva@espol.edu.ec

# Supplementary Figures and Tables

## Supplementary Tables

|  | Df | Sum of Sqs | R^2^ | F | Pr(>F) |
| --- | --- | --- | --- | --- | --- |
| Samples | 4 | 0.75616 | 0.37230 | 0.8606 | 0.5959 |
| Sequencing Platform | 1 | 0.39620 | 0.19507 | 1.8036 | 0.1966 |
| Residual | 4 | 0.87869 | 0.43263 |  |  |
| Total | 9 | 2.03104 | 1.00000 |  |  |

**Table S1.** PERMANOVA based on Bray-Curtis dissimilarities of the effect of the sequencing techniques and the differences between the samples of the different fermentation time points.

| Assembly | 0h | 24h | 48h | 72h | 96h |
| --- | --- | --- | --- | --- | --- |
| # contigs (>= 0 bp) | 1377 | 1077 | 1711 | 2129 | 2471 |
| # contigs (>= 1000 bp) | 1377 | 1077 | 1711 | 2129 | 2471 |
| Total length (>= 0 bp) | 6443423 | 4083071 | 7443463 | 7279303 | 9101217 |
| Total length (>= 1000 bp) | 6443423 | 4083071 | 7443463 | 7279303 | 9101217 |
| # contigs | 1377 | 1077 | 1711 | 2129 | 2471 |
| Largest contig | 209185 | 56373 | 116083 | 105724 | 193233 |
| Total length | 6443423 | 4083071 | 7443463 | 7279303 | 9101217 |
| GC (%) | 41.55 | 40.81 | 44.5 | 53.76 | 46.88 |
| N50 | 8167 | 5669 | 7928 | 4531 | 5922 |
| N90 | 1643 | 1550 | 1534 | 1526 | 1365 |
| L50 | 99 | 175 | 159 | 452 | 292 |
| L90 | 875 | 750 | 1109 | 1542 | 1684 |
| # N's per-100 kbp | 0 | 0 | 0 | 0 | 0 |

**Table S2**. Quast statistics of the hybrid assembly between Nanopore and Illumina sequencing data
